# Use of Ionic Liquid Pretreated and Fermented Sugarcane Bagasse as an Adsorbent for Congo Red Removal

**DOI:** 10.3390/polym13223943

**Published:** 2021-11-15

**Authors:** Uroosa Ejaz, Agha Arslan Wasim, Muhammad Nasiruddin Khan, Othman M. Alzahrani, Samy F. Mahmoud, Zeinhom M. El-Bahy, Muhammad Sohail

**Affiliations:** 1 Department of Microbiology, University of Karachi, Karachi 75270, Pakistan; uroosaejaz24@gmail.com; 2 Department of Biosciences, Shaheed Zulfikar Ali Bhutto Institute of Science and Technology (SZABIST), Karachi 75600, Pakistan; 3 Department of Chemistry, University of Karachi, Karachi 75270, Pakistan; arslan.wasim@uok.edu.pk (A.A.W.); nasiruk@uok.edu.pk (M.N.K.); 4 Department of Biology College of Science, Taif University, P.O. Box 11099, Taif 21944, Saudi Arabia; o.alzahrani@tu.edu.sa; 5 Department of Biotechnology, College of Science, Taif University, P.O. Box 11099, Taif 21944, Saudi Arabia; s.farouk@tu.edu.sa; 6 Department of Chemistry, Faculty of Science, Al-Azhar University, Cairo 11884, Egypt; zeinelbahy@azhar.edu.eg

**Keywords:** adsorbent, cellulase, cellulose, congo red, lignin, sugarcane bagasse

## Abstract

A large amount of industrial wastewater containing pollutants including toxic dyes needs to be processed prior to its discharge into the environment. Biological materials such as sugarcane bagasse (SB) have been reported for their role as adsorbents to remove the dyes from water. In this study, the residue SB after fermentation was utilized for the dye removal. A combined pretreatment of NaOH and methyltrioctylammonium chloride was given to SB for lignin removal, and the pretreated SB was utilized for cellulase production from *Bacillus aestuarii* UE25. The strain produced 118 IU mL^−1^ of endoglucanse and 70 IU mL^−1^ of β-glucosidase. Scanning electron microscopy and FTIR spectra showed lignin and cellulose removal in fermented SB. This residue was utilized for the adsorption of an azo dye, congo red (CR). The thermodynamic, isotherm and kinetics studies for the adsorption of CR revealed distinct adsorption features of SB. Untreated SB followed Langmuir isotherm, whereas pretreated SB and fermented SB obeyed the Freundlich isotherm model. The pseudo-second-order model fitted well for the studied adsorbents. The results of thermodynamic studies revealed spontaneous adsorption with negative standard free energy values. Untreated SB showed a 90.36% removal tendency at 303.15 K temperature, whereas the adsorbents comprised of pretreated and fermented SB removed about 98.35% and 97.70%, respectively. The study provided a strategy to utilize SB for cellulase production and its use as an adsorbent for toxic dyes removal.

## 1. Introduction

Dyes which are used in industries, particularly in the textile sector, possess many health hazards. According to Robinson et al. [1], 2% of the used dyes are dumped directly into the aqueous system. Most of the dyes are toxic, even carcinogenic, and cause adverse effects to aquatic life [2]. Particularly, congo red (CR) is a diazo anionic dye used in many industrial sectors, although its use is banned. This dye can be metabolized to benzidine, which is a known carcinogenic agent [3]. Currently, ozonation, coagulation/flocculation, oxidation, and ultrafiltration are used for the dye removal [4]. All these methods have intense energy requirements, hence, are costly and release hazardous by-products [5]. Therefore, the development of an eco-friendly, efficient, and low-cost technique is needed for dye removal. Adsorption is a noticeable technology among all the available treatment methods because of its low cost and efficiency [6]. The search for inexpensive and efficient adsorbent is mandated by the widespread pollution caused by dyes, particularly by those which are discharged by textile industries.

Nowadays, the world is moving toward the utilization of plant biomass [7]. Sugarcane industries produce around 100 million tons of sugarcane bagasse (SB) worldwide as an agricultural waste by-product [8]. Currently, most of the SB is burnt for energy production, which not only causes environmental problems but also wastes this valuable bio-resource [9] SB is composed of holocellulose, embedded in an amorphous matrix of hemicellulose and lignin [10,11]. Cellulose, along with its other plentiful application, can be utilized for cellulase production by fermentation processes [12]. Worldwide, cellulases are reported to be the third largest group in the enzyme market [13]. At present, cellulases are used in the animal feed, textile, food, paper, and wine industries [14]. However, the pretreatment of SB is required prior to fermentation because of the recalcitrant nature of lignin [15]. Amongst pretreatments by chemical agents, imidazolium-based ionic liquids (ILs) have been widely used. However, these ILs also possess serious risks to the environment as these are not readily biodegradable [16,17]. Alternatively, methyltrioctylammonium chloride has been reported to solubilize lignin by breaking the hydrogen bonds in presence of less toxic solvents at lower temperatures [18,19,20]. So far, native SB has been utilized as an adsorbent to remove toxic dyes from the environment [19], however, the utilization of pretreated and fermented SB have not been reported for this purpose. The literature survey presented several native residues as adsorbents such as orange and banana peel [20], waste bamboo culms [21], calcined bones [22], vegetable residues [23], *Jatropha curcas* pods [24], agricultural waste products [25], coconut shell activated carbon [26], fishery waste [27], coffee grounds [28], tea waste [29], rice hull ash [30], tobacco steam ash [31], modified silica gel [32], and ground eggshell waste [33]. The use of fermented residue in the dye removal can be considered as economical and zero-waste biorefinery.

In this study, SB was pretreated by alkali and IL for lignin removal, and then prereated SB was used for cellulase production by a thermophilic bacterium, *Bacillus aestuarii* UE25 for cellulose removal. The leftover residue after the fermentation was utilized as an adsorbent. The present work is an extensive study on congo red removal by the alkali and ionic liquid pretreated and fermented SB. The adsorption chemistry, thermodynamics, and isotherm kinetics of congo red removal by SB were analyzed.

## 2. Materials and Methods

### 2.1. Lignin Removal from Sugarcane Bagasse

Sugarcane bagasse (SB) was procured from a local sugar industry. It was ground to 300 µ pore size and termed as untreated SB (UTB). It was pretreated sequentially with sodium hydroxide and methyltrioctylammonium chloride (IL) as mentioned by Ejaz et al. [12] and referred as pretreated SB (PTB). Methyltrioctylammonium chloride (≥90%, under the trade name of Aliquat^®^ 336) was purchased from Sigma-Aldrich (Burlington, NJ, USA) and used without any purification. 

### 2.2. Fermentation of Pretreated Sugarcane Bagasse for Cellulose Degradtion

*Bacillus aestuarii* UE25 was purified on nutrient agar (Oxoid, Lenexa, KS, USA). For inoculum preparation, an isolated bacterial colony was transferred to nutrient broth and incubated for 24 h at 60 °C. The density of the inoculum was maintained at 0.3 OD_600_ by using DIATEK ELISA READER DR 200Bc, Wuxi Hiwell Diatek Instruments Co., Ltd, Wuxi, China. Inoculum (7.12%, *v*/*v*) was separately transferred to mineral salt medium (MSM) as described by Mandels and Weber [34] with 1% (*w*/*v*) of PTB, 0.5% (*w*/*v*) of glucose and peptone and incubated at 60 °C with 150 rpm for 48 h. Crude enzyme was obtained by centrifuging the content at 1372× *g* for 10 min and was used to perform enzyme assays by dinitrosalicylic (DNS) method [35]. While left over fermented SB (FTB) was utilized for the dye removal.

### 2.3. Enzyme Assay

Carboxymethyl cellulose and salicin was used as substrate for EG and BGL, respectively. Crude enzyme (25 µL) and 0.5% substrate (25 µL) prepared in 50 mM sodium citrate buffer (pH 4.8) was added in a test tube and kept for 15 min at 60 °C. After the reaction time, DNS reagent (150 µL) was added, and the reaction mixture was boiled for 5 min. Distilled water (720 µL) was added to the mixture, and the absorbance was recorded at 540 nm with heat inactivated enzyme blank [12]. 

### 2.4. Scanning Electron Microscopy and Fourier Transform Infrared (FTIR) Spectroscopy

The structural morphology of UTB, PTB, and FTB was determined by scanning electron microscope (JEOL, Model number: JSM-6380A) (Boston, MA, USA). For FTIR, the samples were heat-dried and coated with KBr. FTIR spectra were recorded with a JASCO FTIR-4200 (Easton, PA, USA).

### 2.5. Adsorption Experiments

Adsorption was conducted by using 50 mL of congo red (CR) solution with known initial concentrations. The adsorbent (SB) dose was 0.1%. The pH of the solutions was adjusted at 4.5, and the mixture was shaken at 50 rpm. For each test, aliquots were filtered using a 0.45 µm membrane filter and CR concentration in the filtrate was determined. 

### 2.6. Concentration of CR and Adsorption Capacity of SB

Congo red (CR) concentration was determined by using a calibration curve as constructed by taking OD_492_ of various solutions. The following equations were used to determine the % removal and the adsorption capacity (*q_t_*):

$$\mathrm{CR}\;removal\left(\%\right)=\frac{\left(C_{i}-C_{t}\right)}{C_{i}}\times 100$$

$$q_{t}=\frac{\left(C_{i}-C_{t}\right)}{m}\times V$$

where *V* is the volume of the dye solution (mL), m is the weight of adsorbent (g), and *C_i_* and *C_t_* are the initial dye concentration (mg L^−1^) and dye concentration (mg L^−1^) in the solution at time t, respectively. In case t adsorption of CR carried out for 18 h at 30 °C, the respective adsorption capacity obtained was termed as *q_e_*, and the dye concentration in solution was termed as *C_e_*.

## 3. Results and Discussion

### 3.1. Pretreatment and Fermentation of Sugarcane Bagasse

**Figure 1:**
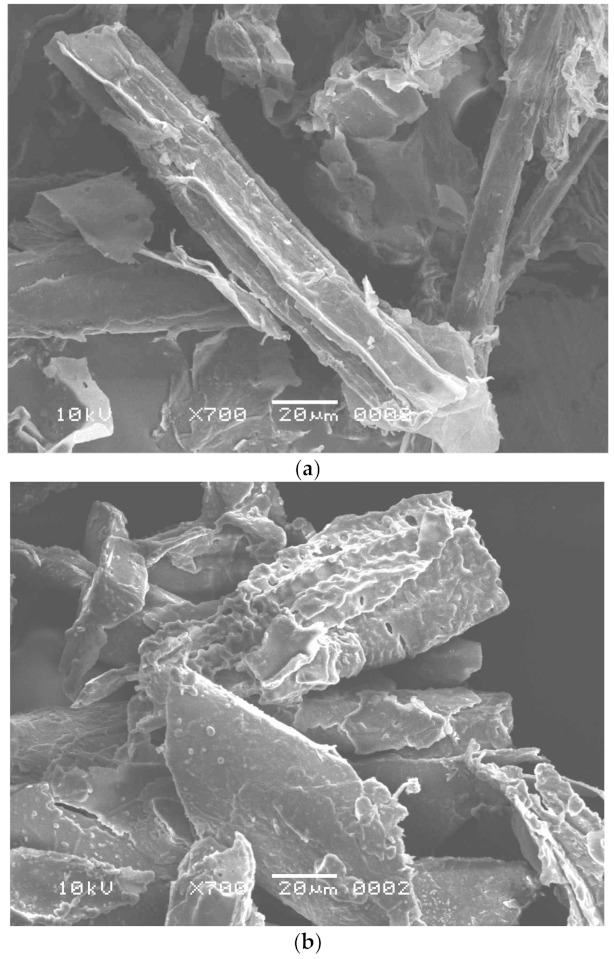

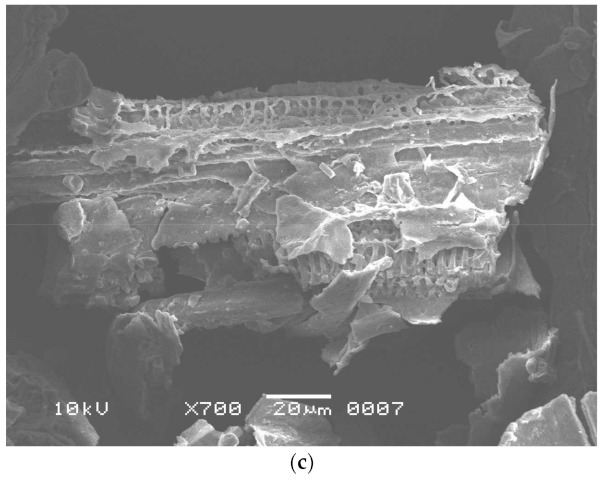
Scanning electron microscopy of (**a**) untreated sugarcane bagasse, (**b**) pretreated sugarcane bagasse, and (**c**) fermented sugarcane bagasse.

Worldwide, sugar mills produce thousands of tons of SB as waste material [36]. In a highly competitive environment, the non-utilization of SB ends up with a loss of resources as it represents a part of the investment that does not generate revenue. Thus, it becomes imperative to examine the possibilities to use this waste product. The purpose of this study was to reuse the waste by-product, SB, for enzyme production and dye removal. This waste product is rich in cellulosic components and may be used as a low-cost energy and carbon source for cellulase production [37] and can also be utilized as an adsorbent for dye adsorption after pretreatment [4] and fermentation. In this study, *B. aestuarii* UE25 fermented PTB and produced 118 IU mL^−1^ of EG and 70 IU mL^−1^ of BGL. The cellulase produced in this study can be used in textile and food industry to obtain valuable industrial product. The effects of pretreatment and fermentation on SB were observed by SEM analysis and compared with the native substrate, which had a smooth surface with no pores (Figure 1a) [38]. The Alkali and IL pretreatment removed lignin from SB and caused physical changes such as the detachment of fibers and a loosening of the matrix (Figure 1b) [39]. So far, many ILs have been investigated to remove lignin from SB [40]. Imidazolium based hydrophilic ILs pose environmental hazards as these are not readily biodegradable [40]. Therefore, in this study, an ammonium-based IL was used to pretreat SB which is water insoluble quaternary ammonium salt [12]. Methyltrioctylammonium chloride also provides an advantage of recyclability as reported earlier [41] where this IL was recycled for six times retaining its 60% pretreatment efficiency. It is clear in Figure 1c that the fibers of SB were destructed after fermentation, which were otherwise absent in the Figure 1a, and hence, showed that the fermentation resulted in efficient cellulosic component removal from the SB [12].

**Figure 2:**
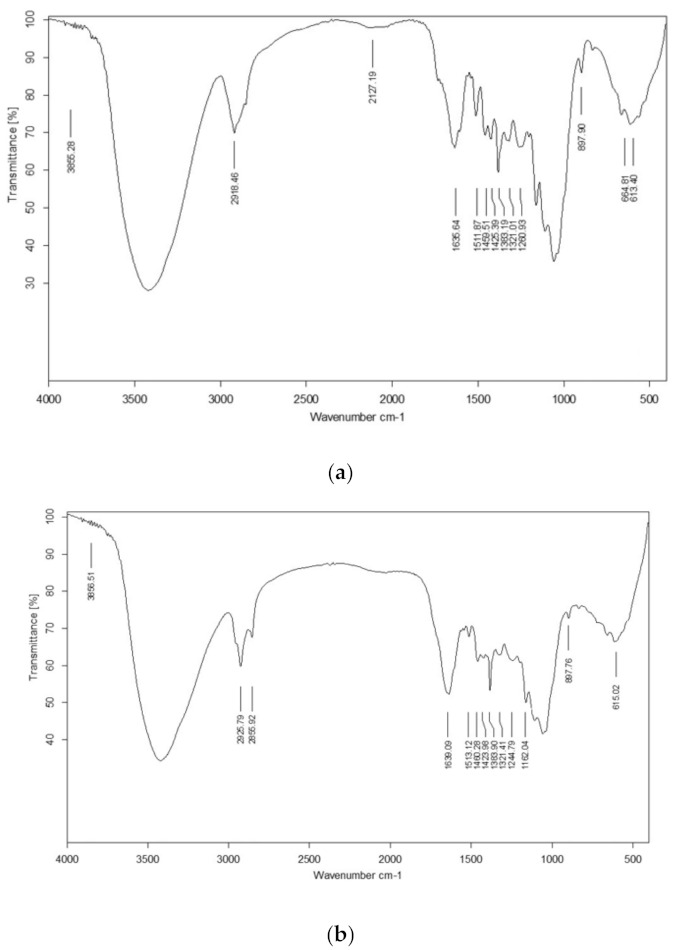
FTIR spectra of (**a**) pretreated sugarcane bagasse and (**b**) fermented sugarcane bagasse.

FTIR spectra were studied for pretreated and fermented substrates (Figure 2) to better understand the changes in lignin and cellulosic content and the data was correlated with the UTB as given by Ejaz et al. [12]. Lignin removal after pretreatment was evident by the peaks in the region of 1260 cm^−1^ and from 1425 cm^−1^ to 1511 cm^−1^ [42]. The noticeable changes in the region associated with lignin moiety was also observed at 3420.48 cm^−1^. The presence of cellulose in the pretreated SB was indicated by the asymmetrical stretching of CH_2_ and CH at 2918 cm^−1^ [12]. In fermented residue, the hydrolysis of cellulose was revealed by changes in the region between 1057 cm^−1^ and 1162 cm^−1^ [12] . Change in cellulosic content in FTB as compare to PTB was highlighted by the changes in the region of 2856 cm^−1^ and 2925 cm^−1^ [12]. The increase in the asymmetry of the curves and line width in the range of 3000 cm^−1^ and 3000 cm^−1^ in the fermented residue indicated the disturbance in the crystalline structure of cellulose [12]. 

### 3.2. Use of Untreated, Pretreated, and Fermented Sugarcane for Dye Removal

Dyes commonly used in textile industries generate considerable amounts of colored wastewater. Congo red (CR) is one out of many azo dyes which are present in textile effluent [43]. The aromatic structures and synthetic origin of these dyes make them non-biodegradable, hence it is difficult to remove them from the textile effluents [44]. The objectives of this study included to assess the suitability of FTB for the adsorption of CR. The literature survey did not turn up with the studies on fermented or pretreated SB for the dye removal. As it is cleared from the Figure 1c that fermentation caused formation of many pores, therefore, it was assumed that fermented residue can serve as a good adsorbent for the dye from the contaminated water. The adsorptive removal was studied at different initial CR concentrations, followed by the adsorption isotherm, kinetics, and thermodynamic studies. 

### 3.3. Effect of Initial Concentration of Dye

**Figure 3:**
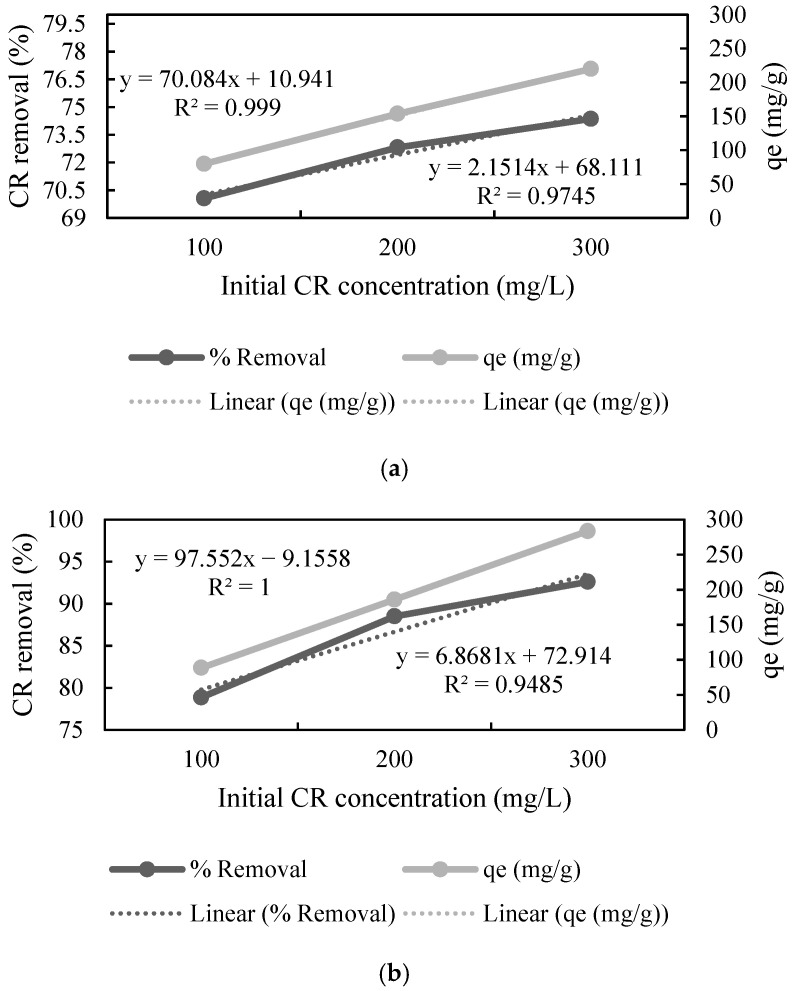

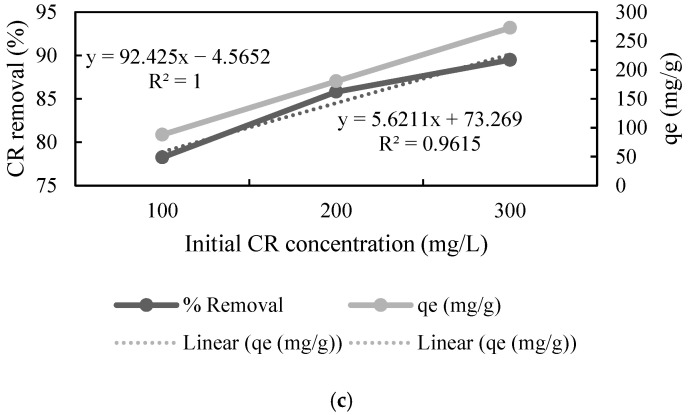
Effect of initial congo red (CR) concentration on adsorption by (**a**) untreated, (**b**) pretreated, and (**c**) fermented sugarcane bagasse (*q_e_* stands for adsorption capacity).

The effect of the initial concentration of the dye was studied by varying it in the range of 100 to 300 mg L^−1^. The adsorption data showed that an increase in the concentration of CR resulted in a decrease in the % removal by UTB, whereas increased concentration of CR increased % removal by PTB and FTB (Figure 3), which means that these substrates can adsorb a greater amount of CR as compared to the native substrate. Furthermore, the adsorption capacity (*q_e_*) increased almost linearly (r^2^ = 0.999, r^2^ = 1 and r^2^ = 1) with the increase in the CR concentration for all the substrates. The highest *q_e_* was 219.892, 283.55, and 272.685 mg L^−1^ for UTB, PTB, and FTB, respectively (Figure 3). The results showed that the pretreatment and fermentation improved the adsorption properties. The direct relationship between the initial CR concentration and *q_e_* concluded that *q_e_* could be further improved by increasing the initial concentration of CR. Zhang et al. [45] also reported that increase in initial dye concentration resulted in better adsorption by ball-milled SB.

### 3.4. Adsorption Isotherm

Adsorption isotherm data were obtained by carrying out the adsorption process at equilibrium. The data were fitted into two isotherm models, namely, the Langmuir and Freundlich isotherm models. The applications of these two adsorption isotherms are described elsewhere [4,32,46]. 

The Langmuir isotherm is expressed as follows:

$$\frac{C_{e}}{q_{e}}=\frac{C_{e}}{q_{m}}+\frac{1}{K_{L}q_{m}}$$

where *K_L_* is the Langmuir constant about the energy of adsorption (1/mg), and *q_m_* is the maximum adsorption capacity with complete monolayer coverage at the adsorbent surface [47,48].

The Freundlich isotherm is expressed as follows:

$$logq_{e}=\frac{1}{n}logq_{c}+logK_{F}$$

where 1/*n* is the adsorption intensity, and *K_F_* (mg^1−1/n^ L^1/n^/g) represents the adsorption capacity [48].

CR’s adsorption by UTB fitted the Langmuir isotherm with r^2^ = 0.999 (Table 1) (Figure S1), which showed the monolayer adsorption of CR onto the surface of this substrate with a finite number of identical sites. The Langmuir isotherm model generally describes gas-solid phase adsorption, which means that adsorption can only occur at definite localized sites [49]. Meanwhile, the adsorption on PTB and FTB fitted well with the Freundlich isotherm model with r^2^ = 0.994 and 0.997 (Table 1) (Figure S2), respectively, which showed that these adsorbents have heterogeneous adsorption sites at their surface [50]. The Freundlich model is known to describe the reversible and non-ideal adsorption, which was not restricted to the formation of monolayer, i.e., it is applied to multilayer adsorption over the heterogenous surface [51].

### 3.5. Adsorption Kinetics

Two models were used to evaluate adsorption kinetics named as the pseudo-first-order and the pseudo-second-order model. The kinetics parameter according to the pseudo-second-order model can be obtained as the slope and intercept value of the plot *t/q_t_* versus *t*. The kinetics studies helped to determine the controlling mechanism of CR adsorption on the UTB, PTB, and FTB. 

The linearized form of the Pseudo-first order equation is as follows:

$$log\left(q_{e}-q_{t}\right)=logq_{e}-\frac{k_{1}t}{2.303}$$

In addition, the linearized form of the pseudo-second-order model is as follows:

$$\frac{t}{q_{t}}=\frac{1}{q_{e}}t+\frac{1}{k_{2}q_{e}^{2}}$$

Here, *q_e_* stands for the quantity of CR dye adsorbed at the equilibrium point, *q_t_* is the amount of CR dye adsorbed at time *t*, and *k* denotes the rate constant. The kinetics data plot showed that adsorption kinetics for all the substrates obeyed the pseudo-second-order model (Figures S3–S5). According to the magnitude of the correlation coefficient (r^2^), the both kinetic models have good enough correlation with higher than 0.8 (Table 2). The predicted *q_e_* values by the pseudo-second-order model were close to experimental *q_e_* (Table 2), which proved the adsorption to be the rate-limiting step. Other studies showed that CR’s adsorption kinetics mostly followed the pseudo-second-order model [31,36]. The successful treatment of the experimental data with kinetic models is necessary to validate the adsorption process.

Intra-particle diffusion kinetic model was also used to analyze CR adsorption by all the types of substrates (Table 3) (Figure S6). The model is expressed as follows:

$$q_{t}=k_{id}t^{0.5}+I$$

where *I* shows the thickness of the boundary layer, and *k_id_* is the intra-particle diffusion rate constant (mg/(g min^0.5^)). For the UTB and PTB, multi-linearity is observed, whereas plot multi-linearity is not prominent for the FTB (Figure S6). Multi-linearity was caused due to the existence of different rate-determining reactions for different time durations. The plot’s steep part corresponded to a rate determining but a relatively faster but short-lived process. The process is usually referred to as the fast coverage of the outer surface of the adsorbent particle. The flat part of the plot corresponded to another rate-determining process established later, which was a slower process. The flat part is attributed to the diffusion through meso/micropores [45].

### 3.6. Thermodynamic Study

The thermodynamics of dye adsorption by SB was studied in a temperature range of 30 to 45 °C. Increasing the temperature from 30 °C to 45 °C caused the reduction in CR removal from 90.36% to 88.32%, 98.35% to 90.09%, and 97.70% to 91.72% for UTB, PTB, and FTB, respectively (Table 4). UTB showed a 90.36% removal tendency at 303.15 K temperature, whereas the adsorbents comprised of PTB and FTB removed about 98.35% and 97.70%, respectively. It reaffirmed that pretreatment and fermentation resulted in the better adsorption tendency than that of the native substrate due to an increase in the surface area upon undergoing different treatments.

Thermodynamic parameters such as the change in entropy (*ΔS˚*), enthalpy (*ΔH˚*), and standard free energy (*ΔG˚*) were determined using the rate equation and the van’t Hoff equation. 

The rate equation is as follows:

$$\Delta G˚=-RTln\;K_{c}$$

and the van’t Hoff equation is as follows:

$$lnK_{c}=\frac{\Delta H^{\circ }}{R}\frac{1}{T}+\frac{\Delta S^{\circ }}{R}$$

*K_c_* is the ratio of the dye’s equilibrium concentration on adsorbent to the equilibrium concentration of the dye in solution. *T* is the adsorption temperature in Kelvin and *R* is the ideal gas constant (8.314 J mol^−1^ K^−1^). Negative values of *ΔG˚* showed the spontaneous nature of the process with a shift of *ΔG˚* from −5.641 to −5.352, −10.315 to −5.839, and −9.45 to −6.362 kJ mol^−1^ for UTB, PTB, and FTB, respectively, when temperature was increased. The trend concludes higher spontaneity at the lower temperatures. The adsorption process was endothermic as concluded by considering positive *ΔH˚* value. No significant changes occurred in the internal structure of adsorbent through the adsorption process. The adsorption process decreased randomness at the solid–solution interface, as revealed by negative *ΔS˚*. It is observed that the extent of adsorption decreased with the increase in temperature; such behavior is typically shown by exothermic adsorption reaction. However, the enthalpy change in the adsorption for the system under study concluded that the adsorption is endothermic. A possible explanation of this anomaly can be given by considering the kinetic energy of adsorbed molecules. The kinetic energy of molecules always increases with the increase in temperature. The increase in kinetic energy overcomes the forces of attraction exerted by the adsorbent surface [52]. The effect is especially prominent in physical adsorption where weak forces hold molecules; therefore, a decrease in temperature results in more adsorption of CR onto the prepared adsorbents. 

## 4. Conclusions

In this study, SB was pretreated by methyltrioctylammonium chloride for lignin removal and cellulosic component of SB was utilized by a thermophilic strain (UE25) of *Bacillus aestuarii* which produced cellulase enzyme. The enzyme obtained in this study can be applied for biotechnological applications. Lignin and cellulose removal in fermented residue were evident by scanning electron microscopy and FTIR. Furthermore, the left-over residue after fermentation appeared as an efficient adsorbent for congo red removal. The adsorbents had surface heterogeneity, as explained by the Freundlich Isotherm model. The adsorption process followed the endothermic pseudo-second-order kinetics. The study provides a cost-effective strategy to utilize SB to produce thermostable cellulase and to remove toxic congo red dye; hence, it strongly supports the idea of reducing environmental pollution.

## Figures and Tables

**Table 1:** Isotherm parameters of congo red adsorption for untreated sugarcane bagasse (UTB), alkali and ionic liquid pretreated sugarcane bagasse (PTB), and fermented sugarcane bagasse (FTB).

Adsorbent	Freundlich			Langmuir		
	K_F_ ^a^	N ^b^	r^2 c^	K_L_ ^d^	q_m_ (mg/g) ^e^	r^2 c^
UTB	8.628	1.345	0.997	0.008	543.589	0.999
PTB	0.026	0.301	0.994	−0.050	−66.83	0.941
FTB	2.639	0.710	0.997	−0.015	−395.926	0.908

^a^ adsorption capacity, ^b^ adsorption intensity, ^c^ correlation coefficient, ^d^ Langmuir constant, ^e^ maximum adsorption capacity.

**Table 2:** Kinetic parameters of congo red adsorption for untreated sugarcane bagasse (UTB), alkali and ionic liquid pretreated sugarcane bagasse (PTB), and fermented sugarcane bagasse (FTB).

Adsorbent	Adsorption Capacity (*q_e_*) (exp) (mg/g)	Pseudo-First Order			Pseudo-Second Order		
		*q_e_*_1_ ^a^	*k*_1_ ^b^	r^2 c^	*q_e_*_2_ ^a^	*k*_2_ ^b^	r^2 c^
UTB	0.12	0.032	0.159	0.833	0.126	6.649	0.999
PTB	0.138	0.048	0.604	0.965	0.141	27.951	0.999
FTB	0.142	0.008	0.224	0.999	0.143	53.312	1

^a^ quantity of dye adsorbed at equilibrium point, ^b^ rate constant, ^c^ correlation coefficient.

**Table 3:** Intra particle diffusion kinetic model of untreated sugarcane bagasse (UTB), alkali and ionic liquid pretreated sugarcane bagasse (PTB), and fermented sugarcane bagasse (FTB).

Adsorbent	*k_id_* ^a^	*I* ^b^
UTB	0.007	0.086
PTB	0.006	0.116
FTB	0.001	0.136

^a^ intra-particle diffusion rate constant, ^b^ thickness of the boundary layer.

**Table 4:** Thermodynamic parameters of congo red (CR) adsorption by untreated sugarcane bagasse (UTB), alkali and ionic liquid pretreated sugarcane bagasse (PTB), and fermented sugarcane bagasse (FTB).

Adsorbent	Temperature (K)	CR Removal (%)	*ΔG*˚ (kJ/mol) ^a^	*ΔH*˚ (kJ/mol) ^b^	*ΔS*˚ (J/(mol K)) ^c^
UTB	303.15	90.363	−5.641	10.18	−15.38
	308.15	88.528	−5.235		
	318.15	88.324	−5.352		
PTB	303.15	98.358	−10.315	102.46	−303.41
	308.15	97.338	−9.222		
	318.15	90.091	−5.839		
FTB	303.15	97.701	−9.45	68.2	−194.99
	308.15	95.006	−7.547		
	318.15	91.722	−6.362		

^a^ standard free energy, ^b^ enthalpy, ^c^ entropy.

## Data Availability

Data associated with this work are given in a supplementary file. Raw data can be obtained from the corresponding author upon a reasonable request.

## Supplementary Materials

The following are available online at https://www.mdpi.com/article/10.3390/polym13223943/s1, Figure S1: Langmuir isotherm plots for (a) untreated sugarcane bagasse, (b) alkali and ionic liquid pretreated sugarcane bagasse, and (c) fermented sugarcane bagasse. Figure S2: Freundlich isotherm plots for (a) untreated sugarcane bagasse, (b) alkali and ionic liquid pretreated sugarcane bagasse, and (c) fermented sugarcane bagasse. Figure S3: Adsorption kinetics for untreated sugarcane bagasse (a) pseudo-first order model, and (b) pseudo-second order model. Figure S4: Adsorption kinetics for alkali and ionic liquid pretreated sugarcane bagasse (a) pseudo-first order model, and (b) pseudo-second order model. Figure S5: Adsorption kinetics for fermented sugarcane bagasse (a) pseudo-first order model, and (b) pseudo-second order model. Figure S6: Intra-particle diffusion model for adsorption of congo red on (a) untreated sugarcane bagasse, (b) alkali and ionic liquid pretreated sugarcane bagasse, and (c) fermented sugarcane bagasse.
