# Supplementary material for: Use of Ionic Liquid Pretreated and Fermented Sugarcane Bagasse as an Adsorbent for Congo Red Removal
Source: Polymers (Basel). 2021 Nov 15;13(22):3943. doi: 10.3390/polym13223943 (PMC8622147; doi:10.3390/polym13223943)
Supplement: Supplementary file 1 [file polymers-13-03943-s001.zip › polymers-1465981-supplementary.pdf]

Use of ionic liquid pretreated and fermented sugarcane bagasse as an adsorbent for congo red removal

Uroosa Ejaz[a,b], Agha Arslan Wasim[c], Muhammad Nasiruddin Khan[c], Othman M. Alzahrani[d], Samy F. Mahmoud[e], Zeinhom M. El-Bahy[f], Muhammad Sohail[a,*]

[a]Department of Microbiology, University of Karachi, Karachi 75270, Pakistan

[b]Department of Biosciences, Shaheed Zulfikar Ali Bhutto Institute of Science and Technology (SZABIST), Karachi-75600, Pakistan

[c]Department of Chemistry, University of Karachi, Karachi 75270, Pakistan

[d]Department of Biology College of Science, Taif University, P.O. Box 11099, Taif 21944, Saudi Arabia

[e]Department of Biotechnology, College of Science, Taif University, P.O. Box 11099, Taif 21944, Saudi Arabia

[f]Department of Chemistry, Faculty of Science, Al-Azhar University, Nasr City 11884, Cairo, Egypt

*Corresponding author's email: msohail@uok.edu.pk

Figure S1. Langmuir isotherm plots for (a) untreated sugarcane bagasse, (b) alkali and ionic liquid pretreated sugarcane bagasse, and (c) fermented sugarcane bagasse.

Figure S2. Freundlich isotherm plots for (a) untreated sugarcane bagasse, (b) alkali and ionic liquid pretreated sugarcane bagasse, and (c) fermented sugarcane bagasse.

Figure S3. Adsorption kinetics for untreated sugarcane bagasse (a) pseudo-first order model, and (b) pseudo-second order model.

Figure S4. Adsorption kinetics for alkali and ionic liquid pretreated sugarcane bagasse (a) pseudo-first order model, and (b) pseudo-second order model.

Figure S5. Adsorption kinetics for fermented sugarcane bagasse (a) pseudo-first order model, and (b) pseudo-second order model.

Figure S6. Intra-particle diffusion model for adsorption of congo red on (a) untreated sugarcane bagasse, (b) alkali and ionic liquid pretreated sugarcane bagasse, and (c) fermented sugarcane bagasse.

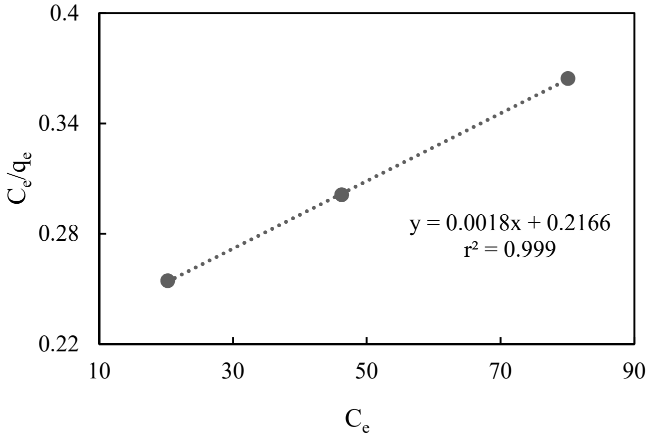
0.4
0.34
0.28
0.22
C_e/q_e
10
30
50
70
90
C_e
y = 0.0018x + 0.2166
r² = 0.999

(a)

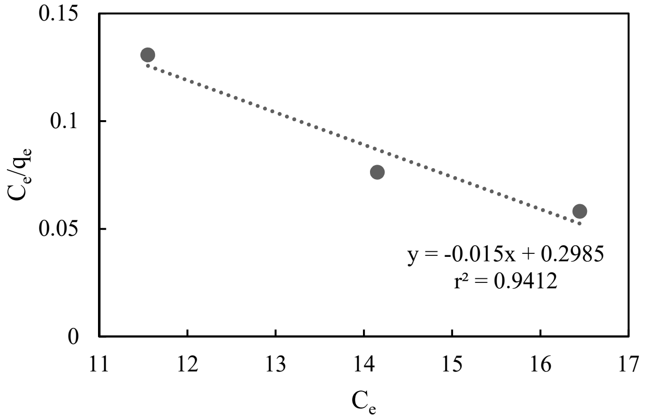
0.15
0.1
0.05
0
C_e/q_e
11
12
13
14
15
16
17
C_e
y = -0.015x + 0.2985
r² = 0.9412

(b)

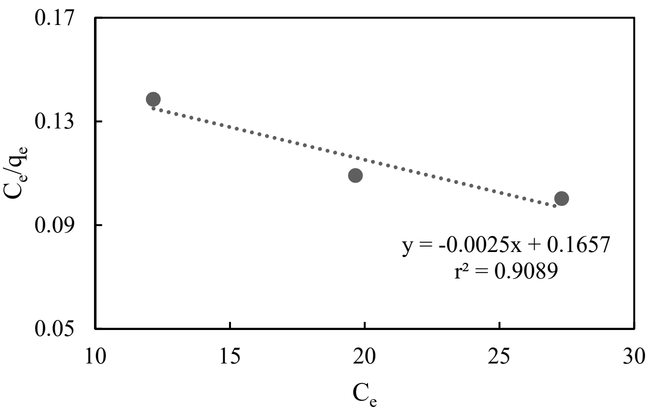

(c)

Figure S1.

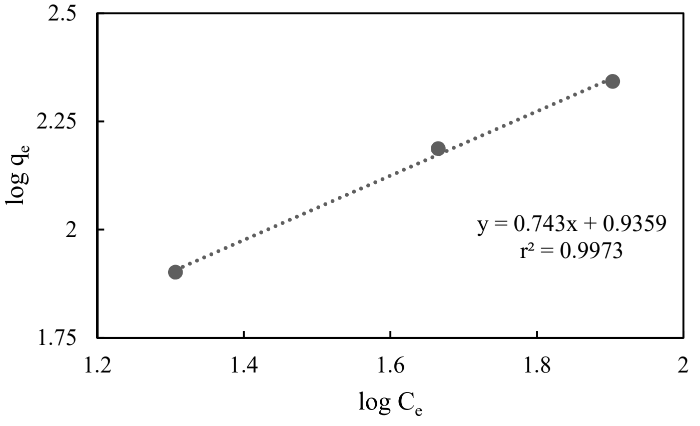
2.5
2.25
2
1.75
log q_e
y = 0.743x + 0.9359
r² = 0.9973
1.2
1.4
1.6
1.8
2
log C_e

(a)

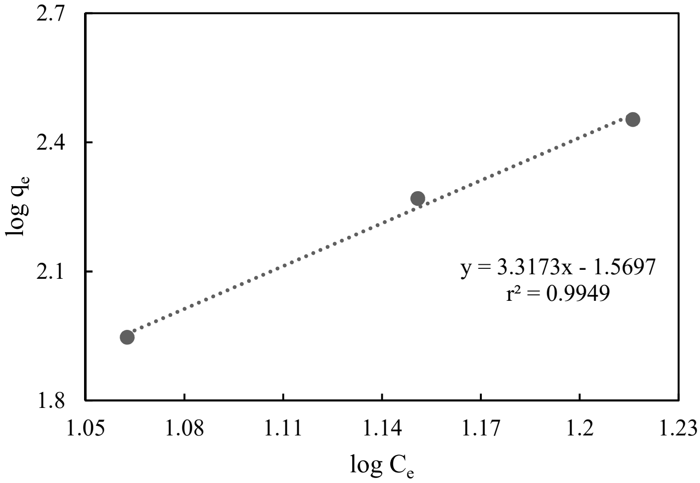
2.7
2.4
2.1
1.8
log q_e
y = 3.3173x - 1.5697
r² = 0.9949
1.05
1.08
1.11
1.14
1.17
1.2
1.23
log C_e

(b)

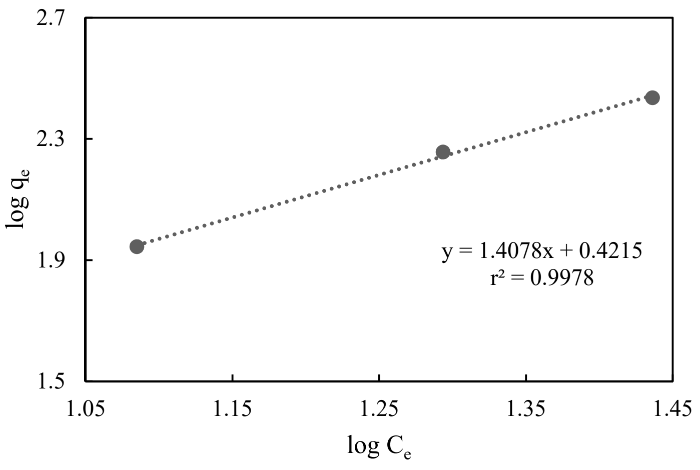
2.7
2.3
1.9
1.5
log q_e
y = 1.4078x + 0.4215
r^2 = 0.9978
1.05
1.15
1.25
1.35
1.45
log C_e

(c)

Figure S2.

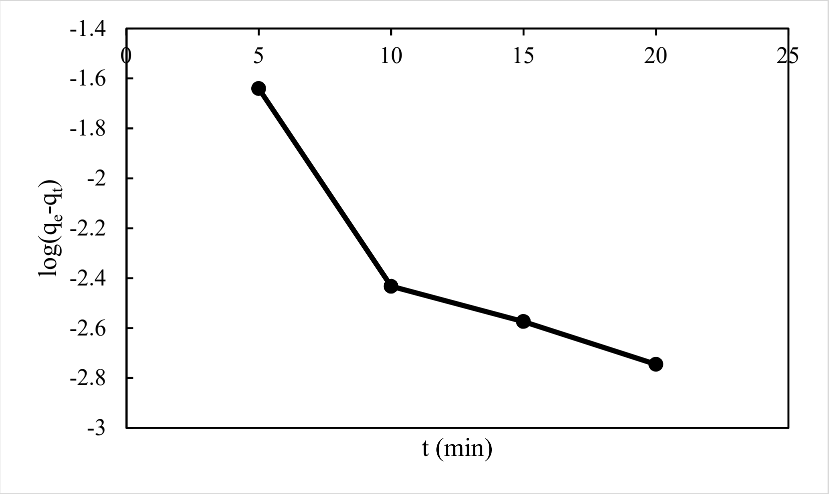

log(q_e-q_t)
t (min)
-1.4
-1.6
-1.8
-2
-2.2
-2.4
-2.6
-2.8
-3
0
5
10
15
20
25

(a)

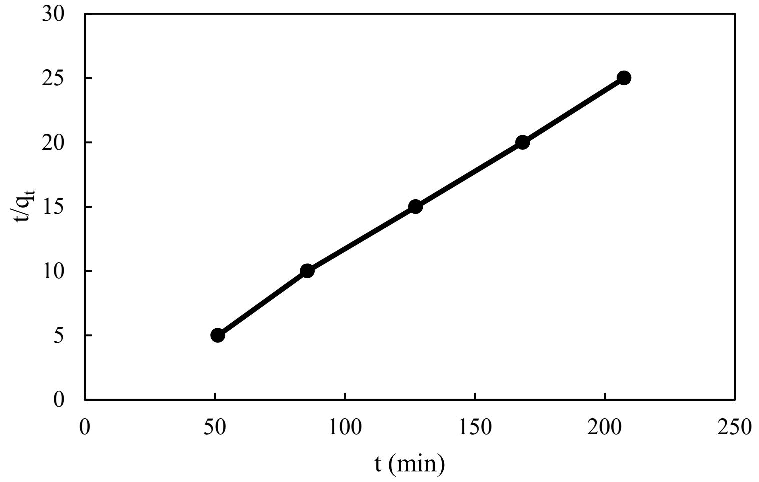

t/q_t
t (min)
30
25
20
15
10
5
0
50
100
150
200
250

(b)

Figure S3.

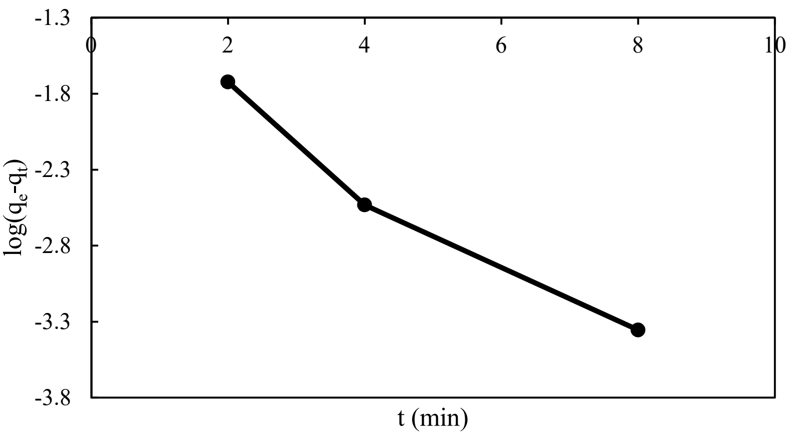

-1.3
-1.8
-2.3
-2.8
-3.3
-3.8
0
2
4
6
8
10
log(q_e-q_t)
t (min)

(a)

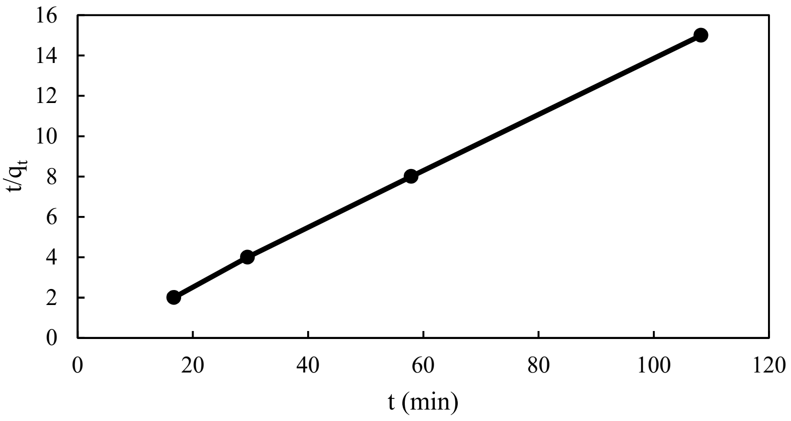

16
14
12
10
8
6
4
2
0
20
40
60
80
100
120
t/q_t
t (min)

(b)

Figure S4.

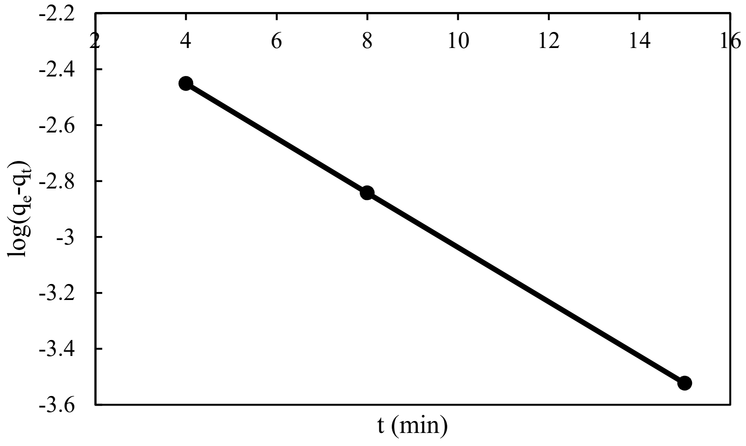

-2.2
-2.4
-2.6
-2.8
-3
-3.2
-3.4
-3.6
2
4
6
8
10
12
14
16
log(qe-qt)
t (min)

(a)

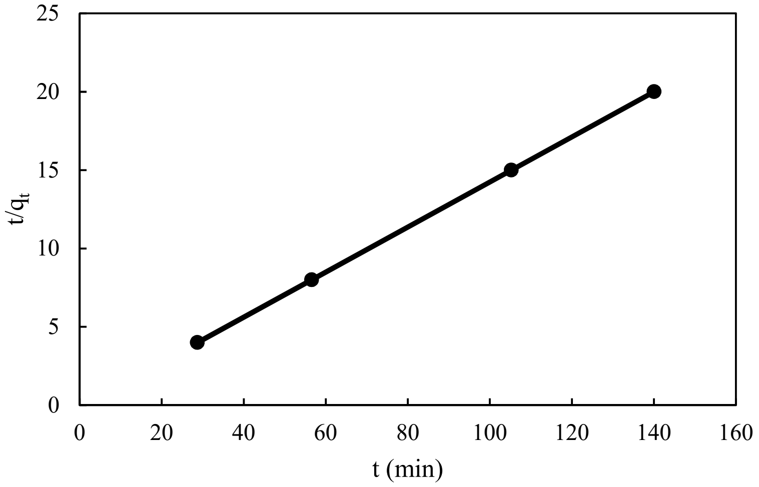

25
20
15
10
5
0
0
20
40
60
80
100
120
140
160
t/qt
t (min)

(b)

Figure S5.

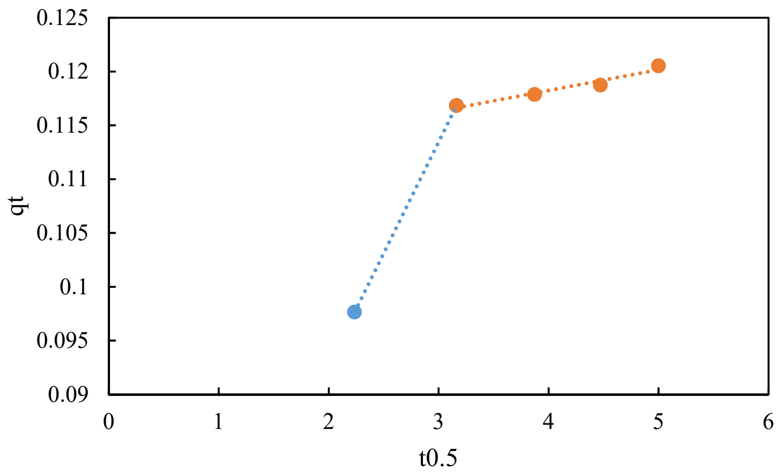
0.125
0.12
0.115
0.11
0.105
0.1
0.095
0.09
qt
0
1
2
3
4
5
6
t0.5

(a)

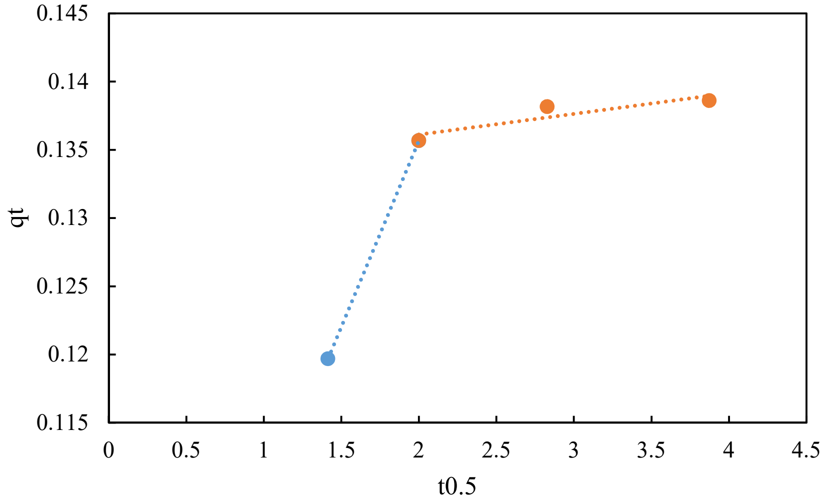
0.145
0.14
0.135
0.13
0.125
0.12
0.115
qt
0
0.5
1
1.5
2
2.5
3
3.5
4
4.5
t0.5

(b)

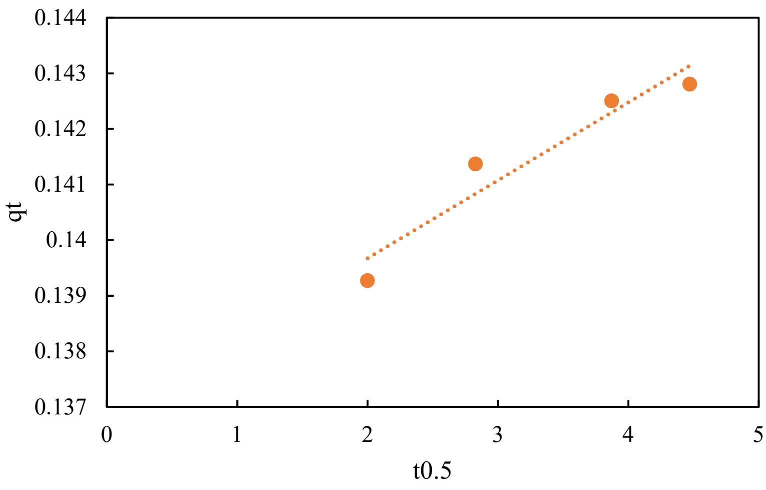

(c)

Figure S6.
